# Hidradenitis suppurativa and rheumatoid arthritis: evaluating the bidirectional association

**DOI:** 10.1007/s12026-021-09221-4

**Published:** 2021-08-19

**Authors:** Khalaf Kridin, Eran Shavit, Giovanni Damiani, Arnon D Cohen

**Affiliations:** 1 Lübeck Institute of Experimental Dermatology, University of Lübeck, Ratzeburger Allee 160, 23562 Lübeck, Germany; 2 Azrieli Faculty of Medicine, Bar-Ilan University, Safed, Israel; 3 Unit of Dermatology and Skin Research Laboratory, Baruch Padeh Medical Center, Poriya, Israel; 4 Dermatology Unit, Barzilai University Medical Center, Ashkelon, Israel; 5 The Faculty of Health Sciences, Ben-Gurion University of the Negev, Beer-Sheba, Israel; 6 Clinical Dermatology, IRCCS Istituto Ortopedico Galeazzi, Milan, Italy; 7 Department of Biomedical, Surgical and Dental Sciences, University of Milan, Milan, Italy; 8 Clalit Health Services, Tel-Aviv, Israel; 9 Siaal Research Center for Family Medicine and Primary Care, Faculty of Health Sciences, Ben-Gurion University of the Negev, Beer Sheva, Israel

**Keywords:** Hidradenitis suppurativa, Rheumatoid arthritis, Risk, Association

## Abstract

Despite some common pathogenic themes, the association of hidradenitis suppurativa (HS) and rheumatoid arthritis (RA) has been poorly investigated. We aimed to evaluate the bidirectional association between HS and RA. A population-based study was conducted to compare HS patients (*n* = 6779) with age-, sex- and ethnicity-matched control subjects (*n* = 33,260) with regard to the incidence of new-onset and the prevalence of preexisting RA. Adjusted hazard ratios (HRs) and adjusted odds ratios (ORs) were estimated. The prevalence of preexisting RA was greater among patients with HS relative to controls (0.5% vs 0.3%. respectively; *p* = 0.019). The odds of being diagnosed with HS were 1.6-fold higher in patients with a history of RA (fully-adjusted OR, 1.66; 95% CI, 1.11–2.49; *p* = 0.014). The incidence rate of new-onset RA was estimated at 4.3 (95% CI, 2.5–6.8) and 2.4 (95% CI, 1.8–3.2) cases per 10,000 person-years among patients with HS and controls, respectively. The risk of RA was comparable between patients with HS and controls (fully-adjusted HR, 1.45; 95% CI, 0.77–2.72; *p* = 0.249). Compared to other patients with HS, those with HS and comorbid RA were older, had a higher prevalence of diabetes mellitus, hypertension, and hyperlipidemia, and had a comparable risk of all-cause mortality. In conclusions, a preexisting diagnosis of RA predisposes individuals to develop HS. Clinicians managing patients with HS and RA should be aware of this association. Further research is required to delineate the underlying pathomechanism of this observation.

**Supplementary Information:**

The online version contains supplementary material available at 10.1007/s12026-021-09221-4.

## Introduction

Hidradenitis suppurativa (HS), previously termed ‘acne inversa’, is a chronic recurrent inflammatory cutaneous disease involving the pilosebaceous unit [1]. HS is characterized clinically by deep-seated nodules, abscesses, and draining sinus tracts that eventually end up as scaring tissue. These are located mostly in the groin, genital areas, and axillae, although the inframammary area, neck, and other anatomical regions may also be affected [2]. The prevalence of HS ranges between 0.05% and 4.0% in different study populations, and it is more frequent in women and smokers [2, 3]. HS typically manifests in the second and third decades of life, and its incidence drops in postmenopausal women, signifying a hormonal influence on the pathogenesis of the disease [4, 5].

Rheumatoid arthritis (RA) is a chronic debilitating disease that manifests as persistent synovitis, which might lead to the destruction of joints and deformation of bones. It affects 0.5–1.0% of western populations, with the prototypical patient being a middle-aged woman. RA has a severe impact on patients’ quality of life owing to its debilitating nature and chronic course [6].

Numerous anecdotal case reports suggested the coexistence of HS and RA in individual patients [7, 8]. However, the epidemiological association between these conditions is yet to be firmly established. In this study, we sought to evaluate the bidirectional association between HS and RA utilizing a large-scale population-based study. We additionally aimed to profile patients with HS and comorbid RA relative to HS patients without RA.

## Methods

### Study design and database

Aiming at elucidating the bidirectional association between HS and RA, the current study followed two study designs. A case–control study that evaluates the prevalence of preexisting RA among patients with HS was conducted to identify the odds of RA in individuals with a history of HS. In addition, a retrospective cohort design that longitudinally follows patients with HS for the incidence of new-onset RA was performed to assess the risk of RA after HS. The study was approved by the institutional review board (IRB) of Ben-Gurion University in accordance with the declaration of Helsinki (approval code: 0212–17-COM).

The current study relied on data retrieved from the computerized database of Clalit Health Services (CHS). CHS is the largest healthcare maintenance organization in Israel, providing a wide assortment of private and public healthcare services for 4,540,768 enrollees as of October 2018, representing 52% of the general Israeli population. The computerized dataset of CHS continuously retrieves data from several sources covering both ambulatory and hospitalized care settings. CHS is additionally typified by free access to healthcare service, comprehensive documentation, and negligible loss to follow-up, which renders its database highly compatible for valid and reliable epidemiological data (8).

### Study population and definition of main variables and covariates

To enroll the current study population, the computerized database of CHS was systematically screened for enrollees with a new-onset diagnosis of HS between January 2000 and December 2018. Patients were subject to inclusion if an (i) HS-specific code was registered by a CHS board-certified dermatologist or (ii) a diagnosis of HS was registered in discharge letters of patients admitted to dermatological inpatient wards [9, 10].

A control group encompassing up to 5 individuals per case of HS was additionally formed. Controls were matched to cases upon sex, age, and ethnicity. Control individuals were enrolled at the same date on which the diagnosis of HS was documented in the corresponding cases. The diagnosis of RA was based on its documentation in the chronic registry of CHS. Cases of RA were defined on the grounds of documentation by board-certified rheumatologists, the purchase of RA-related drugs, and suggestive laboratory and imaging data [11, 12].

Outcome measures were controlled for underlying comorbidities utilizing the Charlson comorbidity index (CCI), an epidemiological scale evaluating the severity of comorbidities among study participants. The latter was proved reliable in predicting mortality (9) and is under widespread usage in epidemiological studies. To avoid overadjustment bias, a modified version of CCI excluding collagen tissue diseases was adopted in the current analysis. Outcome measures were additionally adjusted for body mass index (BMI) and demographic variables. The date of death of study participants was ascertained by linking the study cohort with the National Registry of Deaths Database. In the mortality analysis, all eligible patients were followed up from the onset of HS until December 31st, 2018, or death, whichever occurs earlier.

### Statistical analysis

Baseline characteristics were described by means and standard deviations (SD)s for continuous variables and percentages for categorical variables. The comparison between subgroups was performed using the chi-square test and *t*-test for categorical and continuous variables, respectively.

Logistic regression was used to calculate odds ratios (ORs) and 95% confidence intervals (CIs) to compare cases and control with respect to the presence of preexisting RA. The association was calculated based on individuals who developed HS following the diagnosis of RA in accordance with the presence of a temporal relationship between exposure and outcome in case–control studies.

Incidence rates of RA were calculated for both HS patients and controls and expressed as the number of events per 10,000 person-years. The incidence of these outcomes was calculated merely for individuals without a history of RA prior to the study initiation. Hazard ratios (HRs) and 95% CIs for the risk of new-onset RA were obtained by the use of Cox regression models. The cumulative survival of HS patients with and without RA was calculated using Kaplan–Meier method and compared between the subgroups via stratified log-rank test. Two-tailed *p*-values less than 0.05 were considered statistically significant. All statistical analyses were performed using SPSS software, version 25 (SPSS, Armonk, NY: IBM Corp).

## Results

### Characteristics of the study population

The current study was comprised of 6779 patients with HS and 33,260 age-, sex-, and ethnicity-matched control subjects. The mean (SD) age at the diagnosis of HS was 33.1 (15.1) years, and 4071 (60.1%) were females. The mean BMI and the prevalence of smoking, diabetes mellitus, hyperlipidemia, and hypertension were significantly greater in cases than in controls. The demographic and clinical features of the study participants are outlined in Table 1.

**Table 1:** Descriptive characteristics of the study population

Characteristic	Patients with HS (N = 6779)	Controls (N = 33,260)	*p* value
Age, years			
Mean (SD)	33.1 (15.1)	33.1 (15.1)	0.817
Median (range)	30.0 (0.6–88.7)	30.0 (0.6–88.8)	
Sex, N (%)			
Male	2708 (39.9%)	13,347 (40.1%)	0.780
Female	4071 (60.1%)	19,913 (59.9%)	
Ethnicity, N (%)			
Jews	5607 (82.7%)	27,413 (82.4%)	0.768
Arabs	1172 (17.3%)	5846 (17.6%)	
BMI, mg/kg^2^			
Mean (SD)	27.2 (6.5)	24.7 (5.6)	** < 0.001**
Smoking, N (%)	3591 (53.0%)	11,469 (34.5%)	** < 0.001**
Diabetes Mellitus, N (%)	698 (10.3%)	2236 (6.7%)	** < 0.001**
Hyperlipidemia, N (%)	1972 (29.1%)	7690 (23.1%)	** < 0.001**
Hypertension, N (%)	853 (12.6%)	3220 (9.7%)	** < 0.001**
Charlson comorbidity score			
Mean score (SD)	0.5 (1.2)	0.4 (1.0)	** < 0.001**
None (0)	4891 (72.1%)	26,375 (79.3%)	** < 0.001**
Moderate (1–2)	1469 (21.7%)	5531 (16.6%)	** < 0.001**
Severe (≥ 3)	419 (6.2%)	1355 (4.1%)	** < 0.001**

*HS* hidradenitis suppurativa, *N* number, *SD* standard deviation, *BMI* body mass index

### The odds of hidradenitis suppurativa after rheumatoid arthritis (case–control design)

The prevalence of preexisting RA was greater among patients with HS relative to controls (0.5% vs 0.3%. respectively, *p* = 0.019). Therefore, the odds of being diagnosed with HS were 1.6-fold higher in patients with a history of RA (OR, 1.59; 95% CI, 1.07–2.36). In an age-, sex-, and, ethnicity-stratified analysis, the association of RA with subsequent HS was greater among individuals older than 40 years (OR, 1.73; 95% CI, 1.08–2.79; *p* = 0.022), males (OR, 2.22; 95% CI, 1.01–4.89; *p* = 0.041), and Arabs (OR, 2.93; 95% CI, 1.15–7.45; *p* = 0.018; Table 2). The association between a history of RA and a later diagnosis of HS was only significant in individuals in whom the diagnosis of RA preceded that of HS by more than 10 years (OR, 1.99; 95% CI, 1.09–3.63; *p* = 0.022; Table 2).

**Table 2:** The odds of hidradenitis suppurativa in patients with a preexisting diagnosis of rheumatoid arthritis stratified by age, gender, ethnicity, and latency (case–control study design)

Subgroup	Preexisting RA in patients with HS N(%) (N = 6763)*	Preexisting RA in controls N(%) (N = 33,215)*	OR (95%CI)	Univariate *p* value
All	33 (0.5%)	102 (0.3%)	**1.59 (1.07–2.36)**	**0.019**
Age, years				
< 20	4 (0.3%)	7 (0.1%)	2.80 (0.82–9.59)	0.086
20–40	6 (0.2%)	29 (0.2%)	1.01 (0.42–2.44)	0.976
≥ 40	23 (1.3%)	66 (0.7%)	**1.73 (1.08–2.79)**	**0.022**
Gender				
Male	9 (0.3%)	20 (0.1%)	**2.22 (1.01–4.89)**	**0.041**
Female	117 (2.9%)	438 (2.2%)	1.44 (0.91–2.27)	0.118
Ethnicity				
Jews	26 (0.5%)	90 (0.3%)	1.42 (0.91–2.19)	0.118
Arabs	7 (0.6%)	12 (0.2%)	**2.93 (1.15–7.45)**	**0.018**
Latency after the diagnosis of RA				
0–5 years	7 (0.1%)	29 (0.1%)	1.19 (0.52–2.71)	0.686
6–10 years	11 (0.2%)	36 (0.1%)	1.50 (0.76–2.95)	0.235
> 10 years	15 (0.2%)	37 (0.1%)	**1.99 (1.09–3.63)**	**0.022**
Multivariate analyses				
Age- and sex-adjusted OR			**1.61 (1.08–2.38)**	**0.019**
Fully-adjusted OR**			**1.66 (1.11–2.49)**	**0.014**

*Excluding patients with a diagnosis of RA after HS or recruitment

**Adjusting for age, sex, ethnicity, BMI, and comorbidities

*HS* hidradenitis suppurativa*, RA* rheumatoid arthritis*, OR* odds ratio*, N* number*, CI* confidence interval

*Bold* significant value

We then carried out a multivariate analysis adjusting for putative confounders. The association was robust to a model adjusting for age and sex (age- and sex-adjusted OR, 1.61; 95% CI, 1.08–2.38; *p* = 0.019) as well as to a model adjusting for age, sex, ethnicity, BMI, and comorbidities (fully adjusted OR, 1.66; 95% CI, 1.11–2.49; *p* = 0.014).

### The risk of rheumatoid arthritis in patients with hidradenitis suppurativa (retrospective cohort design)

Patients with HS and controls were cumulatively followed for 37,591.7and 184,763.3 person-years, respectively. During this follow-up duration, the incidence rate of RA was estimated at 4.3 (95% CI, 2.5–6.8) and 2.4 (95% CI, 1.8–3.2) cases per 10,000 person-years in patients with HS and controls, respectively (Table 3).

**Table 3:** Incidence rates and hazard ratio of new-onset rheumatoid arthritis among patients with hidradenitis suppurativa (cohort study design)

	HS (N = 6746)*	Control (N = 33,158)*
Follow-up time, PY	37,591.7	184,763.3
Median follow-up time, years (range)	**6.1 (0.1–18.7)**	**6.1 (0.1–18.7)**
Number of new-onset RA events	16	45
Incidence rate / 10,000 PY (95% CI)	4.3 (2.5–6.8)	2.4 (1.8–3.2)
Unadjusted HR (95% CI) [*p* value]	1.75 (0.99–3.90) [0.055]	Ref.
Male-specific HR (95% CI) [*p* value]	1.79 (0.57–5.62) [0.319]	Ref.
Female-specific HR (95% CI) [*p* value]	1.73 (0.90–3.34) [0.102]	Ref.
< 30 years-specific HR (95% CI) [*p* value]^a^	1.23 (0.35–4.34) [0.753]	Ref.
≥ 30 years-specific HR (95% CI) [*p* value]^a^	**1.94 (1.02–3.69) [0.043]**	Ref.
Jews-specific HR (95% CI) [*p* value]	1.50 (0.79–2.87) [0.216]	Ref.
Arabs-specific HR (95% CI) [*p* value]	3.36 (0.95–11.91) [0.061]	Ref.
Age- and sex-Adjusted HR (95% CI) [*p* value]	1.75 (0.99–3.09) [0.055]	Ref.
Fully adjusted HR (95% CI) [*p* value]^b^	1.45 (0.77–2.72) [0.249]^a^	Ref.

*HS* hidradenitis suppurativa, *RA* rheumatoid arthritis, *HR* hazard ratio*, CI* confidence interval*, PY* person-year

*Excluding patients with a diagnosis of RA before HS or recruitment

^a^Cutoff age was set at 30 since it represents the median age of study participants

^b^Adjusted for age, sex, ethnicity, BMI, and comorbidities (per modified CCI)

*Bold* significant value.

**Fig. 1:**
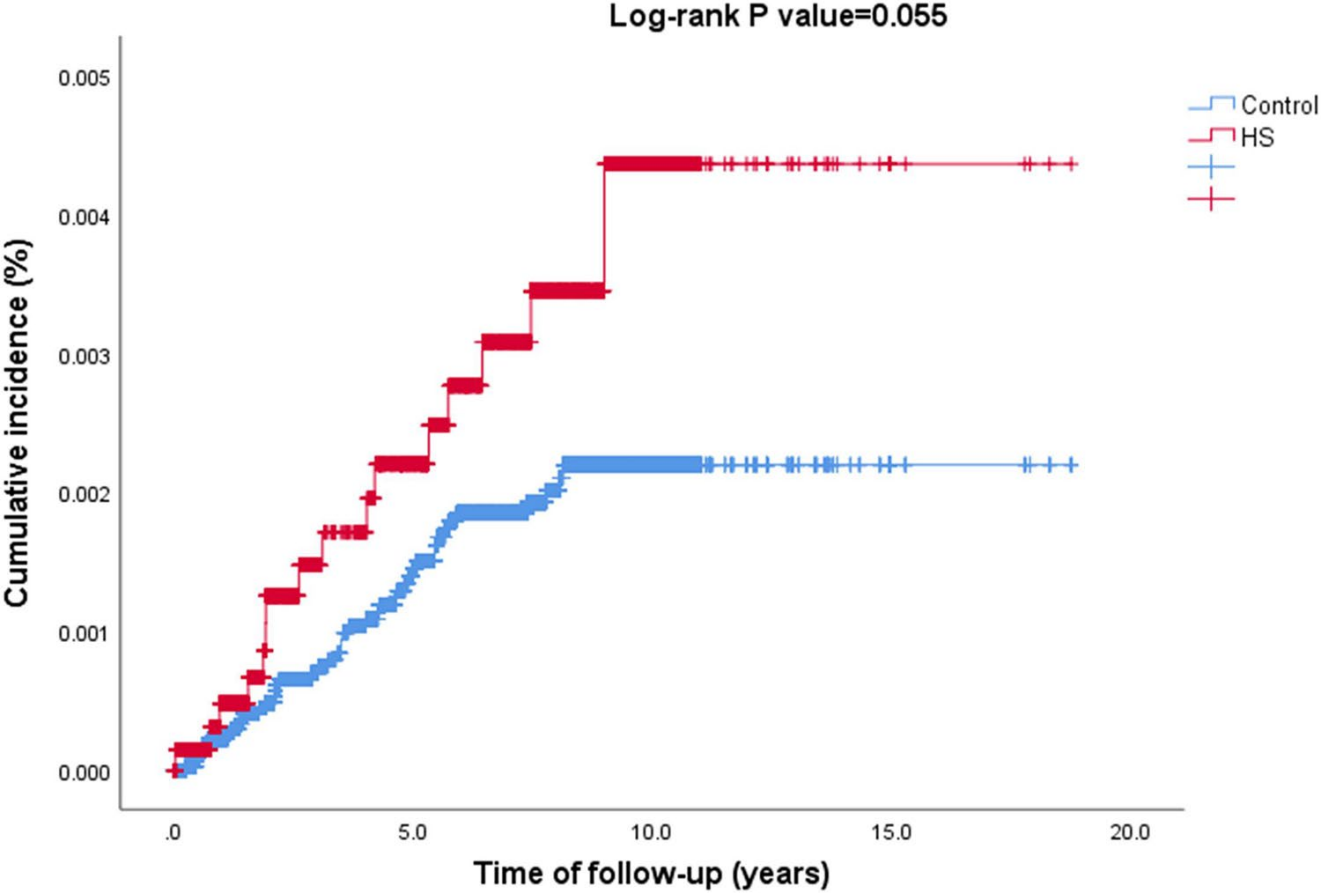
Kaplan Meier curves demonstrating the cumulative incidence of RA among patients with HS and controls

The unadjusted risk of RA was numerically, but not statistically, higher in patients with HS relative to controls (HR, 1.75; 95% CI, 0.99–3.90; *p* = 0.055; Fig. 1). In a stratified analysis, the risk of RA was only elevated in individuals older than 30 years (HR, 1.94; 95% CI, 1.02–3.69; *p* = 0.043). The risk of RA in HS fell short of significance after adjusting for age and sex (age- and sex-adjusted HR, 1.75; 95% CI, 0.99–3.09; *p* = 0.055) as well as for age, sex, ethnicity, BMI, and comorbidities (fully-adjusted HR, 1.45; 95% CI, 0.77–2.72; *p* = 0.249; Table 3).

### Features of patients with hidradenitis suppurativa and comorbid rheumatoid arthritis

The last endpoint of the study was to assess the characteristics of patients with HS and comorbid RA relative to the remaining patients with HS. Patients with HS and comorbid RA were significantly older at the onset of HS, had greater CCI scores, and higher lifetime prevalence of diabetes mellitus, hypertension, and hyperlipidemia (Table 4).

**Table 4:** Characteristics of patients with hidradenitis suppurativa and comorbid rheumatoid arthritis relative to the remaining patients with hidradenitis suppurativa

	Coexistent HS and RA (*n* = 49)	HS without RA (*n* = 6730)	OR	95% CI	*p* value
Age at the onset of HS, years; mean (SD)	48.5 (18.1)	33.0 (15.0)	1.70^a^	1.46–1.98	< 0.001
Female sex, *n* (%)	36 (73.5%)	4035 (60.0%)	1.85	0.98–3.50	0.054
Jewish ethnicity, *n* (%)	38 (77.6%)	5569 (82.7%)	0.72	0.37–1.41	0.338
Body mass index; Kg/m^2^, mean (SD)	34.7 (23.8)	27.5 (20.5)	1.00^b^	0.99–1.01	0.116
Modified Charlson Comorbidity index; mean (SD)	1.3 (2.0)	0.5 (1.1)	1.35^c^	1.18–1.54	< 0.001
Diabetes mellitus, *n* (%)	18 (36.7%)	680 (10.1%)	5.17	2.88–9.28	< 0.001
Hypertension, *n* (%)	18 (36.7%)	835 (12.4%)	4.10	2.28–7.36	< 0.001
Hyperlipidemia, *n* (%)	31 (63.3%)	1941 (28.8%)	4.25	2.37–7.61	< 0.001
Smoking, *n* (%)	26 (53.1%)	3565 (96.9%)	1.00	0.57–1.76	0.990

Change in OR per 10 years^a^, mg/kg^2^ unit^b^ or point in CCI score^c^.

*HS* hidradenitis suppurativa, *RA* rheumatoid arthritis, *OR* odds ratio, *CI* confidence interval, *PY* person-year, *SD* standard deviation

We then evaluated all-cause mortality of patients with HS and comorbid RA as compared to the remaining patients with HS. The risk of all-cause mortality was comparable between the two subgroups (HR, 2.59; 95% CI, 0.64–10.47; *p* = 0.183; *Supplementary figure*).

## Discussion

The current large-scale population-based study revealed that a history of RA confers a 1.6-times increased risk of developing HS. Relative to other patients with HS, those with HS and comorbid RA were significantly older at the onset of HS, had a greater burden of comorbidities, and higher lifetime prevalence of metabolic syndrome.

### The current literature in comparison to our findings

Patients with RA may experience a wide array of cutaneous manifestations [14, 15]. These include, among others, rheumatoid nodules, accelerated rheumatoid nodulosis, rheumatoid vasculitis, palisaded neutrophilic and granulomatosis dermatitis, Felty syndrome, and pyoderma gangrenosum [14, 15]. HS, on the other hand, has been found to coexist with several musculoskeletal diseases, mainly spondyloarthropathies [16, 17]. In their retrospective cohort study following 640 patients with HS, Richette et al. [18] estimated the prevalence of ankylosing spondylitis at 3.7% based on the ESSG criteria, whereas none of their patients was diagnosed with RA.

While the coexistence of HS with RA has been reported anecdotally [7, 8], our knowledge about the association between these conditions is sparse. In a recent population-based study from South Korea, the distribution of comorbidities among patients with HS has been systematically investigated [19]. An increased lifetime prevalence of RA among patients with HS has emerged, yielding a 30% increase in the OR (1.31; 95% CI, 1.16–1.48) relative to control individuals [19]. While the aforementioned study enrolled a larger sample size, its analysis was hampered by a cross-sectional design which did not enable to detect the temporal relationship between the diagnosis of HS and RA. Our study, contrariwise, estimated the bidirectional association of HS by employing two different study designs.

A recent retrospective cohort study followed patients with HS to estimate the risk of developing four types of inflammatory arthritis: ankylosing spondylitis, psoriatic arthritis, other spondyloarthritis, and RA [20]. Following propensity score matching, the risk of RA was significantly increased among patients with HS (HR, 1.16; 95% CI, 1.03–1.31). Given its unidirectional nature, this study was underpowered to investigate the odds of HS after RA [20]. Our study demonstrated a prominent trend towards susceptibility to RA in patients with HS and a significantly increased risk of RA among HS patients older than 30 years of age. Therefore, our findings lend weight, at least to a certain extent, to the aforementioned study [20]. One may assume that this association might have reached the level of statistical significance if a larger sample size was utilized. Further population-based research is warranted to reproduce these findings. Our study denotes that the association of HS with RA was stronger among males. This finding accords with that reported by Lee et al. [19] in their Korean cohort.

### Putative interpretations of the observed association

The pathomechanism underlying the association of HS with RA is yet to be delineated. However, both diseases share some common pathogenic themes. The coexistence of HS and RA was postulated to occur through the exposure of cutaneous antigens that might lead to the deposition of immune complexes in the synovial fluid [21]. Although the final common pathway is different, there are commonalities in the interplay of T and B cell and the activation of proinflammatory cytokines in both diseases, including the overproduction of tumor necrosis factor (TNF) as well as other essential proinflammatory mediators such as interleukin (IL)-6 and IL-1. Therefore, both conditions exhibit a satisfactory clinical response to TNF antagonists [22, 23]. Recent evidence suggested that systemic complement activation might exert a pathogenic role in both HS [24, 25] and RA [26]. Epidemiologically, both diseases are more prevalent in females and cigarette smokers, signifying a predisposing role played by estrogen and nicotine, respectively [27, 28].

### Strengths and limitations

To the best of our knowledge, the current paper represents the first population-based study aiming to evaluate the bidirectional association between HS and RA. The large sample size provides the study with sufficient power to exclude chance as the basis for the findings. Owing to retrieving clinical data from all tiers of healthcare facilities, the study is less susceptible to selection bias than other study designs. The utilization of two study designs enables evaluation of the bidirectional association between the conditions of interests and provides a thorough insight into the temporal relationship between the diseases. Although RA lies in the differential diagnosis of spondyloarthropathies, misclassification in the current study is highly unlikely since the diagnosis of RA relied on documentation by board-certified rheumatologists. Confining cases of HS to those coded by certified dermatologists substantiates the validity of the diagnosis since HS is a clinical diagnosis that should be not mistaken by a trained dermatologist. The current study is not devoid of limitations. Lack of data regarding the clinical characteristics, severity, and immunoserological profile (like seropositivity of anti-CCP and RF antibodies) of patients was the main of these drawbacks.

In conclusion, the current population-based study attested that a history of RA is associated with an elevated risk of subsequent HS. In addition, HS patients older than 30 years are at an increased risk of developing RA. Compared to other patients with HS, those with HS and comorbid RA were significantly older, had a greater burden of comorbidities, and higher lifetime prevalence of metabolic syndrome. Overall survival was comparable between the two subgroups. Our findings emphasize the significance of a multidisciplinary approach as clinicians managing patients with both diseases should be aware of symptoms suggestive of HS and RA and refer patients to specialists at an early stage. In patients with a dual diagnosis of HS and RA, treatment modalities that have been proved effective for both conditions might be preferred. Further research is necessary to delineate the pathomechanism underlying this observation.

## Supplementary Information

Below is the link to the electronic supplementary material.

Supplementary file1 (JPG 152 kb)
