# Supplementary figures and images for: Hidradenitis suppurativa and rheumatoid arthritis: evaluating the bidirectional association
Source: Immunol Res. 2021 Aug 19;69(6):533–40. doi: 10.1007/s12026-021-09221-4 (PMC8580926; doi:10.1007/s12026-021-09221-4)

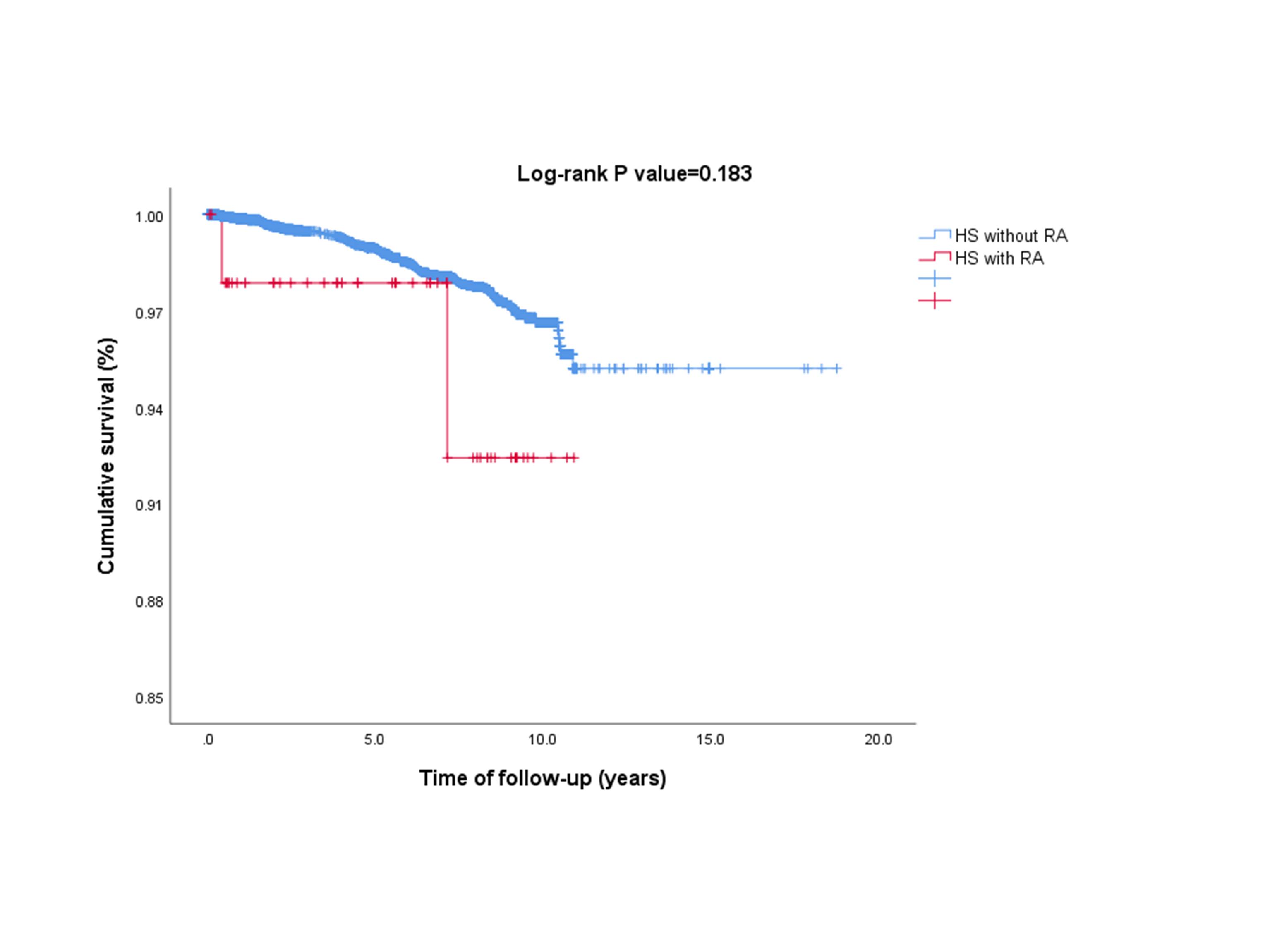

Supplement: Supplementary file 1 — Supplementary file1 (JPG 152 kb) [file 12026_2021_9221_MOESM1_ESM.jpg]
